# Gravid uterine torsion after prone positioning in SARS-CoV2 (COVID-19)-related acute respiratory distress syndrome

**DOI:** 10.1093/jscr/rjac289

**Published:** 2022-06-24

**Authors:** Bavo Hendriks, Evelyne van Uitert, Sam Schoenmakers, Johannes J Duvekot, Diederik Gommers, Jérôme M J Cornette, COVPREG Study Group, Davy van de Sande, Jasper van Bommel, Jeroen J A van Kampen, Jérôme Cornette

**Affiliations:** Department of Uro-Gynaecology, Ghent University Hospital (Universitair Ziekenhuis Gent), Corneel Heymanslaan 10, B-9000 Ghent, Belgium; Department of Obstetrics and Gynaecology, Erasmus MC University Medical Center, Wytemaweg 80, 3015 CN Rotterdam, The Netherlands; Department of Obstetrics and Gynaecology, Erasmus MC University Medical Center, Wytemaweg 80, 3015 CN Rotterdam, The Netherlands; Department of Obstetrics and Gynaecology, Erasmus MC University Medical Center, Wytemaweg 80, 3015 CN Rotterdam, The Netherlands; Department of Obstetrics and Gynaecology, Erasmus MC University Medical Center, Wytemaweg 80, 3015 CN Rotterdam, The Netherlands; Department of Adult Intensive Care, Erasmus MC University Medical Center, Room Ne-413, Doctor Molewaterplein 40, 3015 GD Rotterdam, The Netherlands; Department of Obstetrics and Gynaecology, Erasmus MC University Medical Center, Wytemaweg 80, 3015 CN Rotterdam, The Netherlands; Department of Obstetrics and Gynaecology, Erasmus MC University Medical Center, Wytemaweg 80, 3015 CN Rotterdam, The Netherlands; Department of Adult Intensive Care, Erasmus MC University Medical Center, Room Ne-413, Doctor Molewaterplein 40, 3015 GD Rotterdam, The Netherlands

**Keywords:** uterine torsion, pregnancy, prone positioning, acute respiratory distress syndrome, SARS-CoV2 (COVID-19)

## Abstract

A multiparous pregnant patient was admitted to the intensive care unit in her third trimester of pregnancy for prone positioning mechanical ventilation after developing SARS-CoV2 (COVID-19)-related acute respiratory distress syndrome. Repositioning in left lateral tilt was followed by uterine contractions and cardiotocography alterations. Preterm caesarean section was performed based on persistent foetal tachycardia and suspected foetal distress, followed by a per-operative diagnosis of uterine levotorsion. This case report is the first to explore a potential causal link between prolonged prone positioning in late pregnancy and postural gravid uterine torsion and highlights the need for appropriate foetal monitoring during prone positioning mechanical ventilation support.

## INTRODUCTION

Uterine torsion is a rare condition, defined as ‘the rotation of the uterus of >45 degrees around its longitudinal axis, being dextrorotary in two-thirds of cases’ [1]. Due to its aspecific clinical presentation and often per-operative diagnosis, it has been observed more in the gravid than in the non-gravid uterus. Gravid uterine torsion usually occurs in the third trimester of pregnancy and is associated with increased perinatal mortality rates [1, 2].

Prone positioning is globally being revisited as a safe and effective rescue approach in the management of SARS-CoV2 (COVID-19)-related acute respiratory distress syndrome (ARDS) in pregnancy. It has however not been associated with any form of pelvic organ torsion so far [3, 4]. This case report aims to explore a potential causal link between prolonged prone positioning in late pregnancy and gravid uterine torsion in a multiparous patient.

## CASE REPORT

A 35-year old, gravida-7, para-5, pregnant patient with an uncomplicated obstetric history was admitted to the Intensive Care Unit at a gestational age of 29 weeks and 2 days due to progressive respiratory insufficiency. She tested positive for SARS-CoV2 (COVID-19) 7 days before admission. The patient required a prone positioning ventilation rescue scheme for a total of 5 days, wherein the patient was intermittently turned 4 times from left lateral tilt to prone position. One day after the prone positioning scheme, uterine contractions were observed in the still ventilated patient. Obstetric examination showed a cervical dilation of 3 cm, intact membranes and suboptimal cardiotocography (CTG) alterations with a good foetal condition confirmed through transabdominal ultrasonography. Contractions receded spontaneously and the patient could be extubated 5 days after being repositioned in left lateral tilt.

Foetal condition was monitored on a daily basis throughout the patient’s ICU admission, registering a mean foetal heart rate at 150 beats per minute (bpm). After extubation, a progressive foetal tachycardia developed, with a foetal heart rate rising to 180 bpm, despite a normalizing maternal body temperature and further decreasing C-reactive protein (CRP) levels.

Potential causes for foetal tachycardia were investigated. Foetal anaemia was excluded through doppler ultrasonography (i.e. PSV-ACM 1.5 MoM). Foetal supraventricular tachycardia or other arrhythmias were considered to be less likely as the foetal heart rate remained below 200 bpm with 1:1 atrioventricular conduction, a normal PR interval and no further signs of decompensation. Thyroid function was normal and no stimulant or betamimetic drugs were administered. Maternal arterial blood gas showed no signs of acidosis. As foetal tachycardia increased over the following hours to a baseline of 190–200 bpm, a preterm caesarean section was performed under general anaesthesia at 31 weeks of gestation for suspected foetal distress (Day 20 after diagnosis).

**Figure 1:**
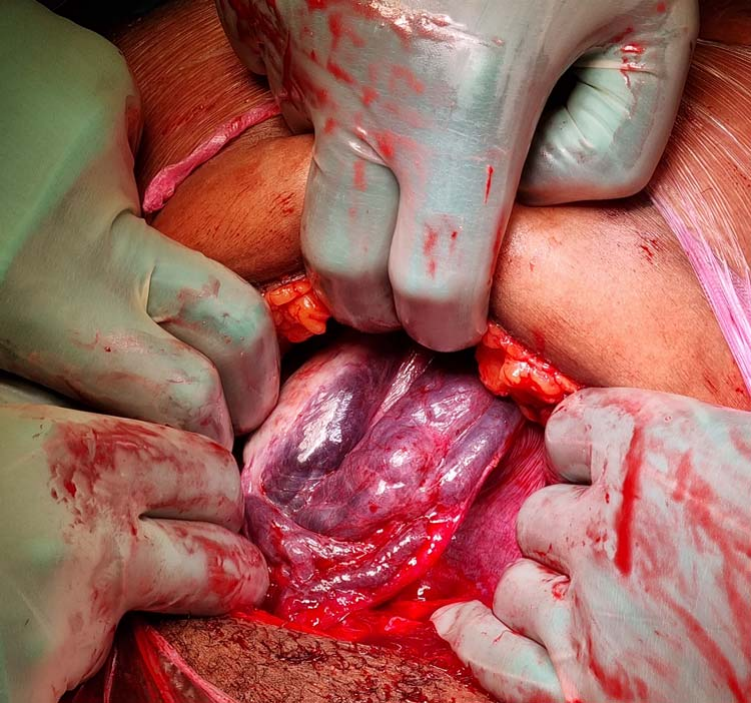
Per-operative diagnosis of uterine levotorsion: right adnexal view with congestion of the para-uterine vascular plexus.

Once the abdominal cavity was reached (Fig. 1), a uterine levotorsion of almost 90 degrees could be observed with a congestion of the para-uterine vascular plexus. No uterine anomalies, leiomyomas or adnexal masses were seen. After uterine repositioning, the operators opted for an anterior, mid-corporeal transverse uterotomy. A girl of 1890 g (89th percentile) was born with a moderate start. The neonate was admitted to the neonatal ICU. Maternal condition rapidly improved after delivery. Clinical examination and ultrasonography showed a normally involuted uterus without any signs of rotation at six weeks postpartum.

## DISCUSSION

Foetal tachycardia is defined as a CTG baseline value >160 bpm for >10 minutes [5]. Maternal pyrexia and inflammatory syndrome are the most frequent causes of foetal tachycardia [5]. SARS-CoV2 (COVID-19)-related ARDS in pregnancy can be associated with a severe maternal inflammatory syndrome but a disease-specific link with foetal tachycardia remains to be established [6]. In retrospect, no clinical signs of perinatal infection or inflammatory syndrome in the newborn could be observed, while no intra- or extra-uterine bacterial agents were further identified. Neonatal polymerase chain reaction (PCR) testing for SARS-CoV2 (COVID-19) came back negative. Placental examination showed signs of diffuse ischaemia yet could not withhold any signs of inflammation or specific fibrin depositions [6, 7].

A potential link between the per-operative diagnosis of gravid uterine torsion and persistent foetal tachycardia was therefore revisited. Parity has been associated with a higher risk of uterine torsion in animal models [8]. Case reports further describe uterine tenderness as a common feature of symptomatic gravid uterine torsion, whereas one case report specificaly describes the onset of isolated foetal tachycardia in an at term, again multiparous patient [1, 2]. In this case, uterine activity and CTG alterations were observed 1 day after the last repositioning of the patient in left lateral tilt. The histological finding of diffuse placental ischaemia could further be matched with the hypothesis raised in previous case reports of gravid uterine torsion having a vasoconstrictory effect on myometrial and placental perfusion [9].

These observations generated the hypothesis of postural uterine torsion through the intermittent repositioning of the patient from prone position to left lateral tilt. The repetitive, dextrorotational movement of ~90 degrees would simultaneously rotate the relaxed para-uterine structures around the longitudinal axis of the uterus. With the weight of the gravid uterus functioning as a gravitational point, this dextrorotational movement would result in a passive uterine levotorsion.

This case report is the first to explore a potential causal link between prolonged prone positioning in late pregnancy and postural gravid uterine torsion in a multiparous patient. As prone positioning mechanical ventilation support has proven to be a safe, effective and often vital rescue method in treating critically ill pregnant patients throughout the COVID-19 pandemic, this case report intends to highlight the need for appropriate foetal monitoring during and after prone positioning in late pregnancy.

## APPENDIX

### COVPREG Study Group

Davy van de Sande MD, Jasper van Bommel (Department of Adult Intensive Care, Erasmus University Medical Center, Rotterdam, The Netherlands), Jeroen J.A. van Kampen MD (Department of Viroscience, Erasmus University Medical Center, Rotterdam, The Netherlands), Jérôme Cornette (Department of Obstetrics and Gynaecology, Erasmus MC University Medical Center, Rotterdam, The Netherlands).
